# Association of neutrophil-to-lymphocyte ratio with stroke morbidity and mortality: evidence from the NHANES 1999–2020

**DOI:** 10.3389/fmed.2025.1570630

**Published:** 2025-04-02

**Authors:** Xin Xu, Guoqiang Zhang, Fei Liu, Jingwei Zheng, Zhijie Jiang, Si Hu, Xudan Shi, Wei Wang, Liang Xu, Zixin Wang

**Affiliations:** ^1^Department of Nursing, The Second Affiliated Hospital of Zhejiang University School of Medicine, Hangzhou, China; ^2^Department of Neurosurgery, Second Affiliated Hospital, School of Medicine, Zhejiang University, Hangzhou, China; ^3^Clinical Research Center for Neurological Diseases of Zhejiang Province, Hangzhou, China; ^4^State Key Laboratory of Transvascular Implantation Devices, Hangzhou, China; ^5^Neuroscience Intensive Care Unit, Second Affiliated Hospital of Zhejiang University School of Medicine, Hangzhou, China; ^6^Department of Neurosurgery, Affiliated Huzhou FuYin Hospital of Huzhou University, Huzhou, China; ^7^Department of Anesthesiology, Second Affiliated Hospital, School of Medicine, Zhejiang University, Hangzhou, China

**Keywords:** cross-sectional study, stroke, NHANES, neutrophil-to-lymphocyte ratio, predictor

## Abstract

**Background:**

Stroke is closely linked to inflammation, with the neutrophil-to-lymphocyte ratio (NLR) emerging as a promising inflammatory marker. This study aims to investigate the association between NLR and both morbidity and mortality in stroke patients.

**Methods:**

Data from the National Health and Nutrition Examination Survey (NHANES) 1999–2020 were analyzed, including adults with complete neutrophil and lymphocyte count records. Multivariate logistic regression was used to examine the relationship between NLR and both stroke morbidity and all-cause mortality. Restricted cubic spline regression was employed to assess potential nonlinearity in these associations. Subgroup analyses were performed to identify influencing factors.

**Results:**

After adjusting for confounders, the adjusted odds ratios (ORs) and 95% confidence intervals (CIs) for stroke in the higher NLR quartiles, compared to the lowest quartile, were 1.28 (1.07–1.52) and 1.36 (1.12–1.65), respectively. The restricted cubic spline curve indicated a nonlinear positive association between NLR and stroke risk. Additionally, an elevated NLR was positively associated with an increased risk of all-cause mortality.

**Conclusion:**

The findings underscore the potential use of NLR in stratifying and predicting mortality risk in stroke patients, suggesting its relevance in clinical practice.

## Introduction

Inflammation represents a complex host response to danger signals and is fundamental for host survival, while also being implicated in the pathogenesis of many human diseases (1). Neuroinflammatory responses play a critical role in the pathophysiology of ischemic stroke, where brain ischemia-induced immunosuppression may promote concurrent infections (2, 3). Inflammatory biomarkers have been widely investigated as potential predictors of cardiovascular diseases. The neutrophil-to-lymphocyte ratio (NLR), a simple ratio derived from routine complete blood count tests, reflects the relative number of neutrophils and lymphocytes in peripheral circulation and serves as a novel biomarker of baseline inflammatory response (4, 5). Previous studies have demonstrated that NLR is associated with poor functional outcomes in patients with acute intracerebral hemorrhage and subarachnoid hemorrhage, and its relevance to stroke is increasingly recognized (5, 6).

Stroke remains the second leading cause of death globally, contributing significantly to disability, with a rising incidence among younger and middle-aged populations (7). Stroke has a complex pathophysiology, including both ischemic and hemorrhagic types, with inflammation playing a critical role in their onset, progression, and outcomes (8). Current consensus indicates that inflammation has a cumulative negative impact on stroke incidence, highlighting it as a crucial therapeutic target for stroke prevention (9). Neutrophils, a type of white blood cell involved in the initial immune response, contribute to inflammation and tissue damage during the acute phase of stroke (10, 11). Similarly, lymphocytes play a vital role in the inflammatory process and are closely associated with the detrimental effects of stroke. In mice deficient in T and B cells, the infarct size was significantly reduced following transient focal ischemia (12). Therefore, the NLR may serve as a surrogate marker for the underlying inflammatory status in patients at risk of stroke.

Research exploring the relationship between NLR and stroke has revealed several significant findings. Elevated NLR levels are associated with an increased risk of stroke, particularly ischemic stroke. This relationship is thought to result from the pro-inflammatory state indicated by a higher neutrophil count and a relatively lower lymphocyte count (5, 13). In the context of ischemic stroke, vascular occlusion leads to hypoxia and nutrient deprivation in brain tissue, exacerbating tissue damage through neutrophil-mediated inflammation (14). Thus, an elevated NLR may reflect a heightened inflammatory response, leading to a worse prognosis among stroke patients (15, 16).

NLR has been investigated as a prognostic indicator in stroke, with higher NLR being associated with poorer outcomes, including increased mortality and greater severity of neurological deficits. These findings suggest that NLR may not only help identify individuals at higher risk of stroke but also predict outcomes in patients who have already experienced a stroke. It holds potential as a simple and cost-effective tool for risk stratification and guiding therapeutic decisions in clinical settings.

Given the rise in stroke morbidity with advancing age, population growth and aging may contribute to substantial increases in global deaths and disabilities in the future (7, 17). There is an urgent need to identify prognostic parameters that are easy to measure and cost-effective to strengthen the monitoring of stroke morbidity and related mortality. The NLR shows promise as a biomarker in the context of stroke, offering insights into the inflammatory processes underlying this complex disease. Its ability to predict stroke risk and outcomes, combined with its simplicity and cost-effectiveness, makes it an attractive tool for both research and clinical practice. Further research is needed to better understand the mechanistic links between NLR and stroke and to explore its potential role in personalized approaches for stroke prevention and management. Comprehensive studies could help elucidate disease mechanisms and identify potential therapeutic targets. The aims of this study were to investigate the association of NLR with stroke morbidity and mortality and to assess the importance of inflammatory status in the development of stroke.

## Materials and methods

### Study population

We utilized data from the 1999–2020 cycles of the National Health and Nutrition Examination Survey (NHANES). This survey is managed by the Centers for Disease Control and Prevention (CDC) and the National Center for Health Statistics (NCHS). NHANES is a complex, multistage probability sampling survey conducted biennially to evaluate the health and nutritional status of the U.S. population, as well as disease profiles, to inform public health policy. The survey method includes face-to-face interviews, physical examinations, and laboratory testing. The study protocol received approval from the NCHS Ethics Review Board, and all participants provided written informed consent prior to participation. Detailed study design and data for NHANES can be accessed on the CDC’s official website.^1^

**Figure 1:**
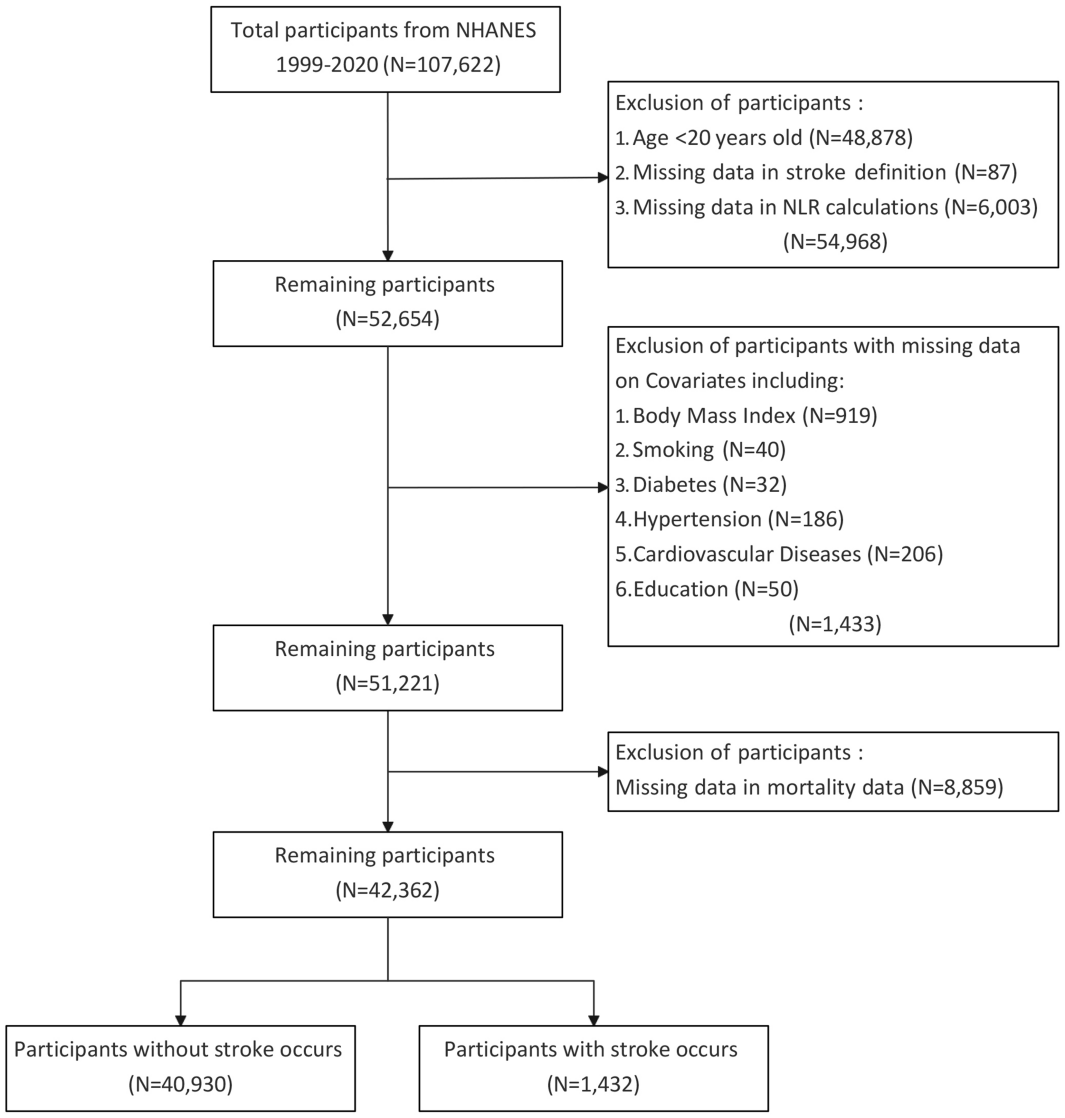
Flowchart of participant selection.

We downloaded data covering the years 1999–2020 from the NHANES website, encompassing 11 survey cycles. The data included in this study comprised demographic, examination, laboratory, and questionnaire information. The initial dataset included cross-sectional data from 107,622 participants. Exclusion criteria were applied to participants lacking complete stroke data and blood cell counts, as well as those under 20 years of age. Those lacking data on covariates were also excluded. After these exclusions, the study ultimately comprised 42,362 eligible subjects for subsequent analysis. A detailed flow chart of the participant recruitment process was shown in Figure 1.

### Assessment of stroke

Stroke patients were identified based on self-reported health status and medical history. Participants were classified as having had a stroke if they responded “yes” to the question, “Has a doctor or other health professional ever told you that you had a stroke?” This question was administered by trained interviewers using a Computer-Assisted Personal Interviewing (CAPI) system, which included built-in consistency checks to reduce data entry errors and employed on-screen help tools to clarify key terms. Alerts were immediately triggered when responses were flagged as unusual, inconsistent, or unrealistic, prompting interviewers to verify or edit the initial responses. It is important to note that self-reported measures are susceptible to recall bias, which may impact data interpretation.

### Measurement of NLR

Neutrophil and lymphocyte counts were determined by trained medical professionals at Mobile Examination Centers using a Beckman Coulter automated hematology analyzer. Complete blood counts were performed on blood samples and reported as ×10 (3) cells/μL. The NLR was calculated by dividing the absolute neutrophil count by the absolute lymphocyte count.

### Assessment of mortality

The study endpoint was all-cause mortality. Mortality data were determined through probabilistic matching between NHANES and the National Death Index (NDI) death certificate records, using personal identifiers such as name, race, gender, date of birth, and social security number. Follow-up duration was defined as the time from the NHANES interview date to the date of death or December 31, 2021.

### Covariates

Various covariates were included in our analysis to account for potential confounders. These covariates, selected based on existing literature, encompassed factors such as age, gender, race, education level, body mass index (BMI), smoking status, diabetes status, hypertension status, congestive heart failure status, and coronary heart disease status. Gender was categorized as female or male. Race/ethnicity was classified as Mexican American, non-Hispanic Black, non-Hispanic White, other Hispanic, or other race/multiracial. Education level was grouped into three categories: less than high school, high school, and more than high school. Diabetes, hypertension, congestive heart failure, and coronary heart disease were defined based on self-reported questionnaire data. Laboratory data, including white blood cell, monocyte, neutrophil, and lymphocyte counts, were obtained from the NHANES website.

### Statistical analysis

Continuous variables were presented as means ± standard deviations (SD), while categorical variables were shown as frequency counts and percentages. *p*-values for continuous variables were calculated using a weighted logistic regression model, and *p*-values for categorical variables were obtained using a weighted chi-square test. NLR was divided into four quartiles, with the first quartile (Q1) designated as the reference quartile.

Kaplan–Meier (K–M) survival analysis was performed, and comparisons were made across NLR quartiles. A multivariable Cox regression model was used to assess the linear associations between NLR and both stroke morbidity and all-cause mortality. Three Cox regression models with varying levels of confounder adjustment were established: Model 1 with no covariate adjustments, Model 2 adjusted for age, gender, and race, and Model 3 adjusted for age, gender, race, education level, BMI, hypertension, diabetes, coronary heart disease, and smoking status. Additionally, a restricted cubic spline regression model was applied to explore potential relationships between NLR and both stroke morbidity and all-cause mortality. Subgroup analyses were conducted to evaluate the impact of NLR on stroke morbidity and all-cause mortality across different subgroups, stratified by age, gender, race/ethnicity, BMI, education level, smoking status, diabetes, hypertension, and coronary heart disease. All statistical tests were two-tailed, with a *p*-value <0.05 considered statistically significant. Statistical analyses were performed using R software version 4.3.1 (R Foundation for Statistical Computing, Vienna, Austria).

## Results

### Baseline characteristics

A total of 51,221 participants were included in the analysis, with a weighted mean age of 49.49 years. The overall stroke prevalence among all participants was 3.68%. Compared to individuals without stroke, stroke patients were generally older and more likely to be non-Hispanic White. They were also less educated, more likely to be smokers, and had higher prevalences of diabetes, hypertension, congestive heart failure, and coronary heart disease. Additionally, stroke patients exhibited higher white blood cell counts, monocyte counts, neutrophil counts, and NLR, coupled with lower lymphocyte counts (all *p* < 0.05). Detailed baseline characteristics of all participants, grouped by stroke status, were presented in Table 1.

**Table 1:** Weighted comparison in basic characteristics.

Characteristic	Overall *N* = 51,221	Stroke *N* = 1,886	Non-stroke *N* = 49,335	*p*-value
Age (years), mean ± SD	49.49 ± 17.99	66.28 ± 13.32	48.85 ± 17.84	<0.001
Gender, %				0.385
Female	52.01	51.01	52.04	
Male	47.99	48.99	47.96	
Race/ethnicity, %				<0.001
Mexican American	17.05	10.39	17.3	
Non-Hispanic Black	21	27.25	20.76	
Non-Hispanic White	44.01	50.32	43.77	
Other Hispanic	8.489	5.779	8.592	
Other race or multi-racial	9.455	6.257	9.577	
Education level, %				<0.001
High school	23.2	26.88	23.05	
Less than high school	26.01	35.79	25.64	
More than high school	50.79	37.33	51.31	
BMI (kg/m^2^), mean ± SD	29.05 ± 6.86	29.76 ± 6.76	29.03 ± 6.86	<0.001
Smoking, %	45.28	59.7	44.73	<0.001
Diabetes, %				<0.001
Borderline	2.054	3.34	2.005	
No	85.87	65.38	86.65	
Yes	12.08	31.28	11.34	
Hypertension, %	34.65	75.24	33.1	<0.001
Congestive heart failure, %				<0.001
Do not know	0.158	0.742	0.136	
No	96.73	82.24	97.28	
Yes	3.112	17.02	2.58	
Coronary heart disease, %	4.121	18.13	3.586	<0.001
WBC (1,000 cells/μL)	7.27 ± 3.00	7.45 ± 2.52	7.26 ± 3.02	0.001
Monocyte (1,000 cells/μL)	0.56 ± 0.20	0.60 ± 0.24	0.56 ± 0.20	<0.001
Neutrophils (1,000 cells/μL)	4.30 ± 1.79	4.51 ± 1.76	4.29 ± 1.79	<0.001
Lymphocyte (1,000 cells/μL)	2.16 ± 2.02	2.07 ± 1.34	2.17 ± 2.04	<0.001
NLR	2.21 ± 1.23	2.53 ± 1.62	2.20 ± 1.21	<0.001

BMI, body mass index; NLR, neutrophil-to-lymphocyte ratio. Mean ± SD for continuous variables: *p*-value was calculated by weighted logistic regression model. % for categorical variables: *p*-value was calculated by weighted chi-square test.

Table 2 summarized the baseline characteristics of participants stratified by NLR quartiles. The mean NLR values for quartiles Q1, Q2, Q3, and Q4 were 1.15, 1.75, 2.31, and 3.75, respectively. Compared to those in the highest quartile, participants with lower NLR were more likely to be younger, female, non-Hispanic Black, have a lower BMI, be nonsmokers, and be free from diabetes, hypertension, congestive heart failure, coronary heart disease, and stroke.

**Table 2:** Baseline demographic and clinical data of NLR four quantiles.

	Q1 *N* = 10,620	Q2 *N* = 11,593	Q3 *N* = 9,602	Q4 *N* = 10,547	Overall *N* = 42,362	*p*-value
NLR	1.15 ± 0.25	1.75 ± 0.16	2.31 ± 0.18	3.75 ± 1.46	2.22 ± 1.22	<0.001
Age (years)	47.22 ± 17.05	48.10 ± 17.30	49.20 ± 18.00	51.72 ± 19.63	49.03 ± 18.08	<0.001
Gender, %						0.006
Female	53.21	52.42	51.74	50.95	52.1	
Male	46.79	47.58	48.26	49.05	47.9	
Race/ethnicity, %						<0.001
Mexican American	16.03	19.45	19.62	17.07	18.04	
Non-Hispanic Black	35.34	17.46	14.77	12.35	20.06	
Non-Hispanic White	31.56	45.22	49.85	56.58	45.68	
Other Hispanic	8.183	8.945	8.165	7.244	8.154	
Other race or multi-racial	8.889	8.928	7.592	6.751	8.073	
BMI (kg/m^2^)	28.46 ± 6.35	28.76 ± 6.38	29.17 ± 6.97	28.95 ± 7.13	28.82 ± 6.70	<0.001
Smoking, %	43.03	43.78	45.9	50.53	45.75	<0.001
Diabetes, %	10.34	10.54	12.32	13.79	11.7	<0.001
Hypertension, %	31.46	31.14	33.66	37.78	33.45	<0.001
Congestive heart failure, %	1.968	2.26	2.739	5.082	2.998	<0.001
Coronary heart disease, %	2.533	3.269	4.103	6.04	3.963	<0.001
Stroke, %	2.533	2.864	3.531	4.665	3.38	<0.001
WBC (1,000 cells/μL)	6.39 ± 2.86	6.93 ± 1.80	7.46 ± 1.95	8.38 ± 2.66	7.28 ± 2.47	<0.001
Monocyte (1,000 cells/μL)	0.53 ± 0.23	0.55 ± 0.18	0.56 ± 0.18	0.59 ± 0.21	0.56 ± 0.20	<0.001
Neutrophils (1,000 cells/μL)	2.94 ± 0.96	3.90 ± 1.07	4.64 ± 1.27	5.90 ± 2.17	4.32 ± 1.80	<0.001
Lymphocyte (1,000 cells/μL)	2.67 ± 2.21	2.23 ± 0.61	2.02 ± 0.55	1.65 ± 0.51	2.15 ± 1.26	<0.001

BMI, body mass index; NLR, neutrophil-to-lymphocyte ratio. Mean ± SD for continuous variables: *p*-value was calculated by weighted logistic regression model. % for categorical variables: *p*-value was calculated by weighted chi-square test.

### Association between NLR and stroke morbidity

**Figure 2:**
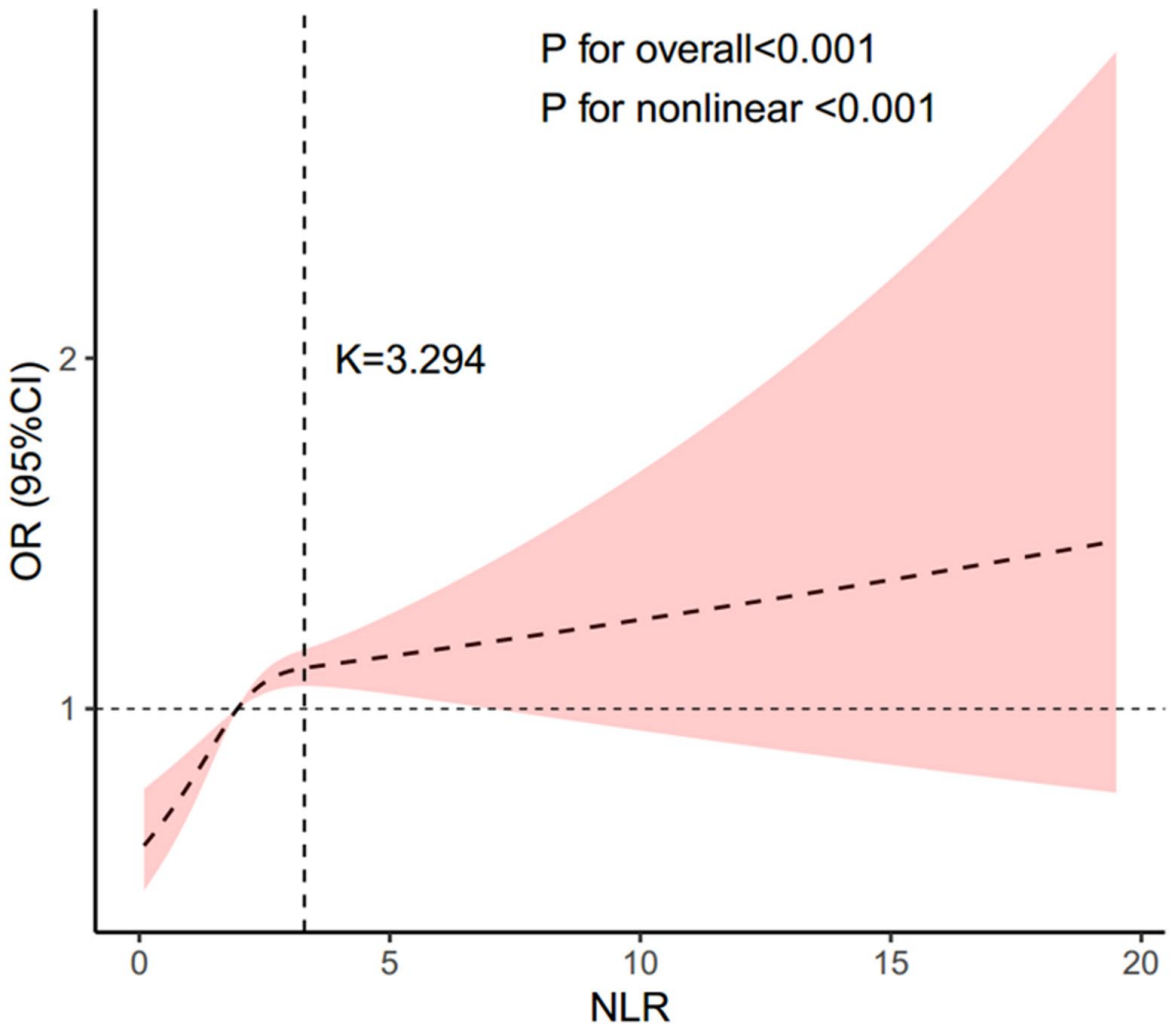
Association between the NLR and stroke morbidity. Adjusted for age, gender, race, educational level, BMI, hypertension, diabetes, coronary heart disease, and smoking status. The solid line and pink area represent the estimated values and their corresponding 95% CIs, respectively. NLR, neutrophil-to-lymphocyte ratio.

In all multivariable models adjusted for potential confounders, NLR was found to be independently and positively associated with stroke morbidity (Table 3). After fully adjusting for confounders, the adjusted odds ratios (ORs) and 95% confidence intervals (CIs) for stroke morbidity in the higher NLR quartiles, compared to the lowest quartile, were 1.12 (0.9–1.4), 1.4 (1.14–1.72) and 1.31 (1.07, 1.6), respectively. The restricted cubic spline regression analysis further substantiated a nonlinear positive association between NLR and stroke risk (*p* for nonlinearity <0.001, Figure 2). Table 4 showed the threshold effect analysis of NLR on stroke morbidity.

**Table 3:** Association between NLR and stroke morbidity.

	Model 1		Model 2		Model 3	
	OR (95% CI)	*p*-value	OR (95% CI)	*p*-value	OR (95% CI)	*p*-value
Continuous	1.17 (1.13, 1.21)	<0.001	1.08 (1.04, 1.12)	<0.001	1.05 (1.01, 1.09)	0.013
Q1	Reference	—	Reference	—	Reference	—
Q2	1.1 (0.89, 1.35)	0.374	1.12 (0.91, 1.38)	0.288	1.12 (0.9, 1.4)	0.293
Q3	1.49 (1.23, 1.81)	<0.001	1.45 (1.18, 1.78)	<0.001	1.4 (1.14, 1.72)	0.002
Q4	1.89 (1.58, 2.28)	<0.001	1.46 (1.2, 1.78)	<0.001	1.31 (1.07, 1.6)	0.008
*p* for trend	<0.001		<0.001		0.005	

Model 1: No covariates were adjusted. Model 2: Age, gender, and race were adjusted. Model 3: Age, gender, race, educational level, BMI, hypertension, diabetes, coronary heart disease, and smoking status.

**Table 4:** Threshold effect analysis of NLR on stroke morbidity.

NLR	Adjusted *β* (95% CI), *p*-value
Model 1	
A straight-line effect	1.085 (1.055–1.116) <0.001
Model 2	
Fold points (*K*)	3.294
< *K* segment effect	1.243 (1.163–1.328) <0.001
> *K* segment effect	1.014 (0.966–1.060) 0.548
Logarithmic likelihood ratio test	<0.001
95% CI of the cut point	1.065, 1.170

**Figure 3:**
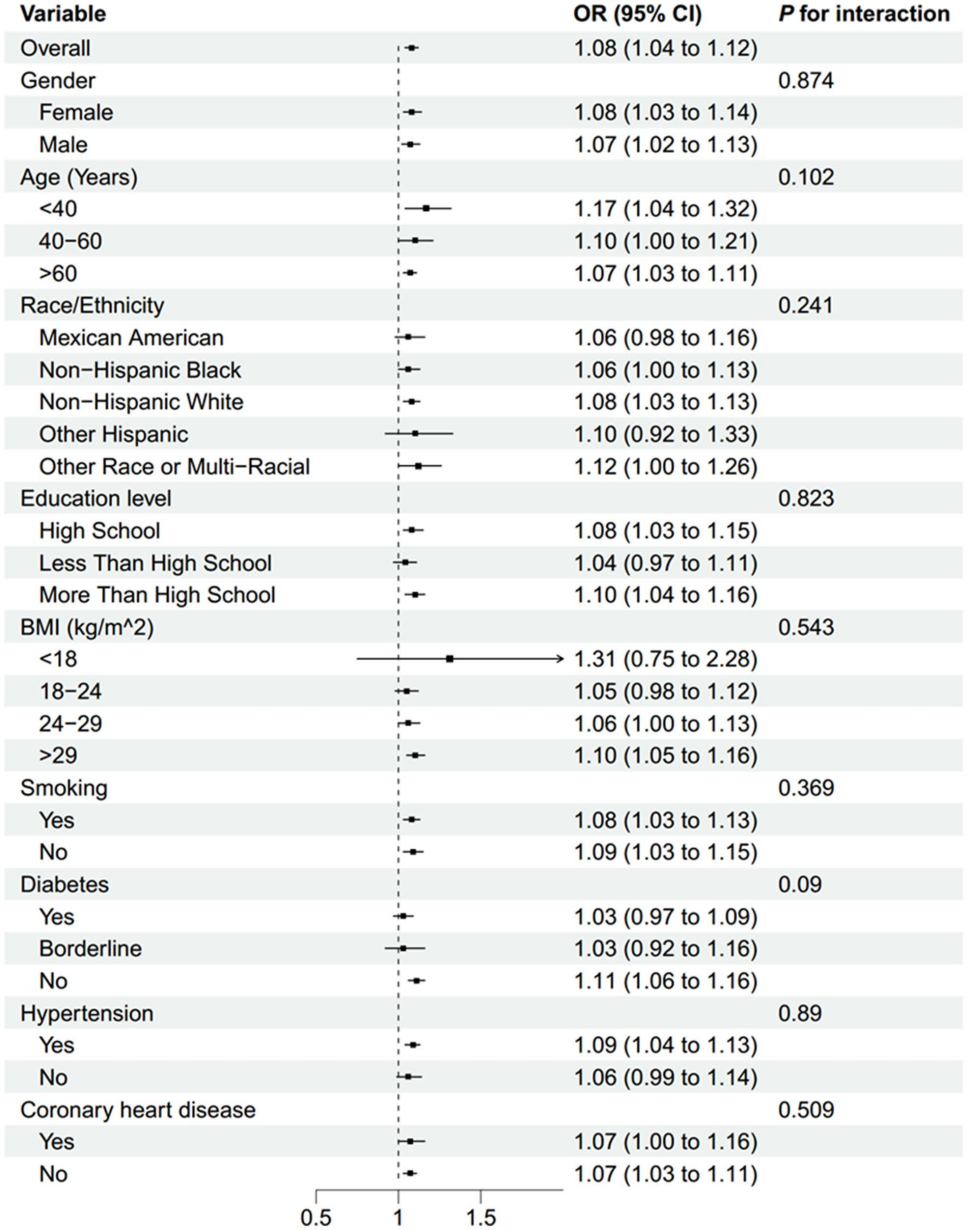
Subgroup analysis of the association between NLR and stroke morbidity. BMI, body mass index; NLR, neutrophil-to-lymphocyte ratio.

Subgroup analyses indicated that the association between NLR and stroke morbidity remained consistent across all stratified variables, including gender, age, race/ethnicity, education level, BMI, smoking status, diabetes, hypertension, and coronary heart disease. No effect modification was observed (all interaction *p*-values >0.05) (Figure 3). Subgroup analyses were conducted to evaluate whether recent infections or inflammatory events modified the association between NLR and stroke morbidity. The results indicated that the association remained consistent across all infectious-associated variables (Supplementary Figure 1).

### Association between NLR and all-cause mortality

Table 5 presented the three Cox regression models used to evaluate the association between NLR and all-cause mortality, as well as the Cox regression model specifically for all-cause mortality among stroke patients. A total of 7,062 deaths were observed among the 42,362 participants. Among these, 740 mortalities occurred in the 1,432 stroke patients included in this subset. After full adjustment for potential confounders, NLR was positively associated with an increased risk of all-cause mortality in stroke patients. Specifically, patients in the fourth NLR quartile had a significantly higher risk of all-cause mortality compared to those in the second quartile (HR, 1.56; 95% CI, 1.2–2.02; *p* < 0.001).

**Table 5:** Cox regression models for the association between the NLR and all-cause mortality.

	Model 1		Model 2		Model 3		
	HR (95% CI)	*p*-value	HR (95% CI)	*p*-value	HR (95% CI)	*p*-value	
All people							
Continuous	1.19 (1.18, 1.2)	<0.001	1.12 (1.11, 1.13)	<0.001	1.12 (1.1, 1.13)	<0.001	7,062
Q1	Reference	—	Reference	—	Reference	—	1,275
Q2	1.04 (0.94, 1.15)	0.41	0.99 (0.9, 1.09)	0.801	1 (0.91, 1.1)	0.967	1,414
Q3	1.16 (1.06, 1.27)	0.001	1.01 (0.92, 1.11)	0.775	1 (0.91, 1.1)	0.973	1,659
Q4	2.17 (1.99, 2.37)	<0.001	1.55 (1.43, 1.68)	<0.001	1.51 (1.4, 1.63)	<0.001	2,714
*p* for trend	<0.001		<0.001		<0.001		
People with stroke							
Continuous	1.14 (1.11, 1.18)	<0.001	1.12 (1.08, 1.15)	<0.001	1.11 (1.08, 1.15)	<0.001	740
Q1	Reference	—	Reference	—	Reference	—	155
Q2	0.96 (0.71, 1.3)	0.798	1 (0.76, 1.3)	0.983	1 (0.76, 1.31)	0.976	164
Q3	1.21 (0.94, 1.54)	0.135	1.13 (0.9, 1.42)	0.298	1.11 (0.89, 1.38)	0.367	181
Q4	2.01 (1.52, 2.66)	<0.001	1.63 (1.24, 2.13)	<0.001	1.56 (1.2, 2.02)	<0.001	240
*p* for trend	<0.001		<0.001		<0.001		

**Figure 4:**
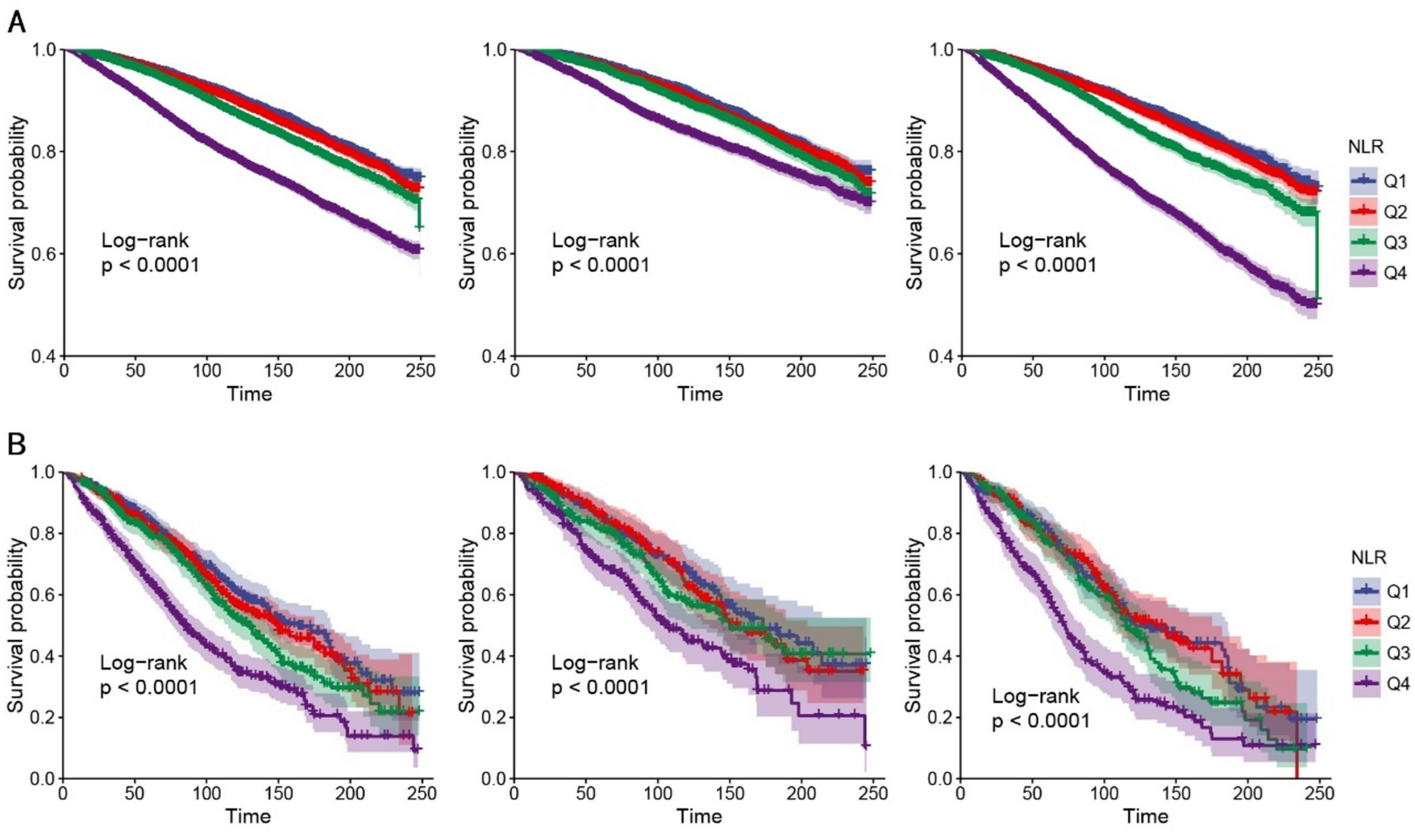
K–M analyses for all-cause mortality among the four groups. **(A)** K–M analyses for all-cause mortality in all people (left), female (middle), and male (right). **(B)** K–M analyses for all-cause mortality in people with stroke (left), female (middle), and male (right). NLR, neutrophil-to-lymphocyte ratio, Q1–Q4, quartiles 1–4.

Kaplan–Meier survival analysis demonstrated significant differences in all-cause mortality across the four NLR quartiles during follow-up, with the highest mortality rate observed in the fourth quartile (log-rank *p* < 0.001) (Figure 4).

**Figure 5:**
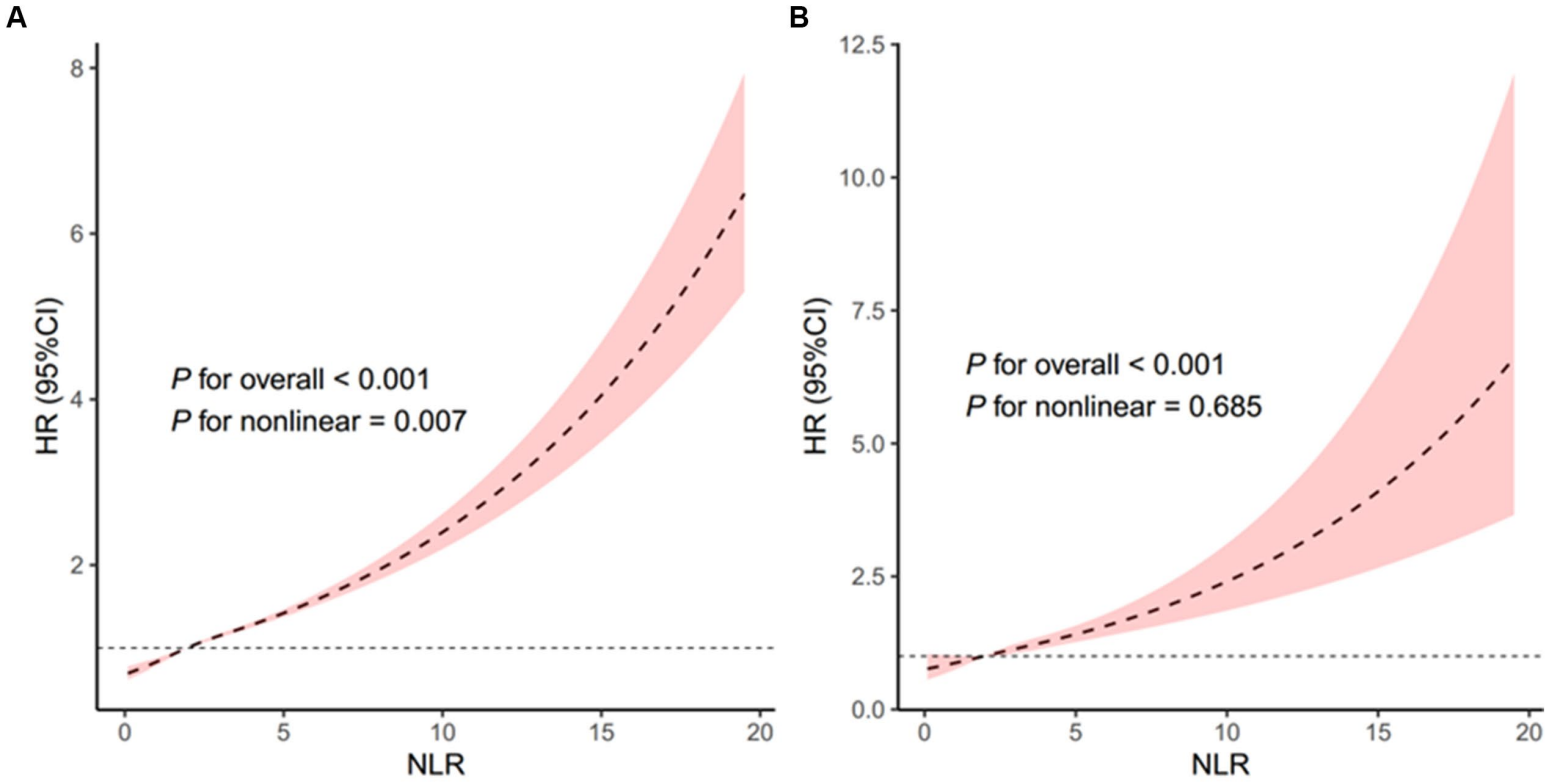
Association of the NLR with all-cause mortality in all people **(A)** and patients with stroke **(B)**. Adjusted for age, sex, and race. The solid line and pink area represent the estimated values and their corresponding 95% CIs, respectively. NLR, neutrophil-to-lymphocyte ratio.

The restricted cubic spline regression analysis further indicated a nonlinear positive association between NLR and all-cause mortality in the overall population (*p* for nonlinearity = 0.007, Figure 5A) and a linear positive association between NLR and all-cause mortality among stroke patients (*p* for nonlinearity = 0.685, Figure 5B).

**Figure 6:**
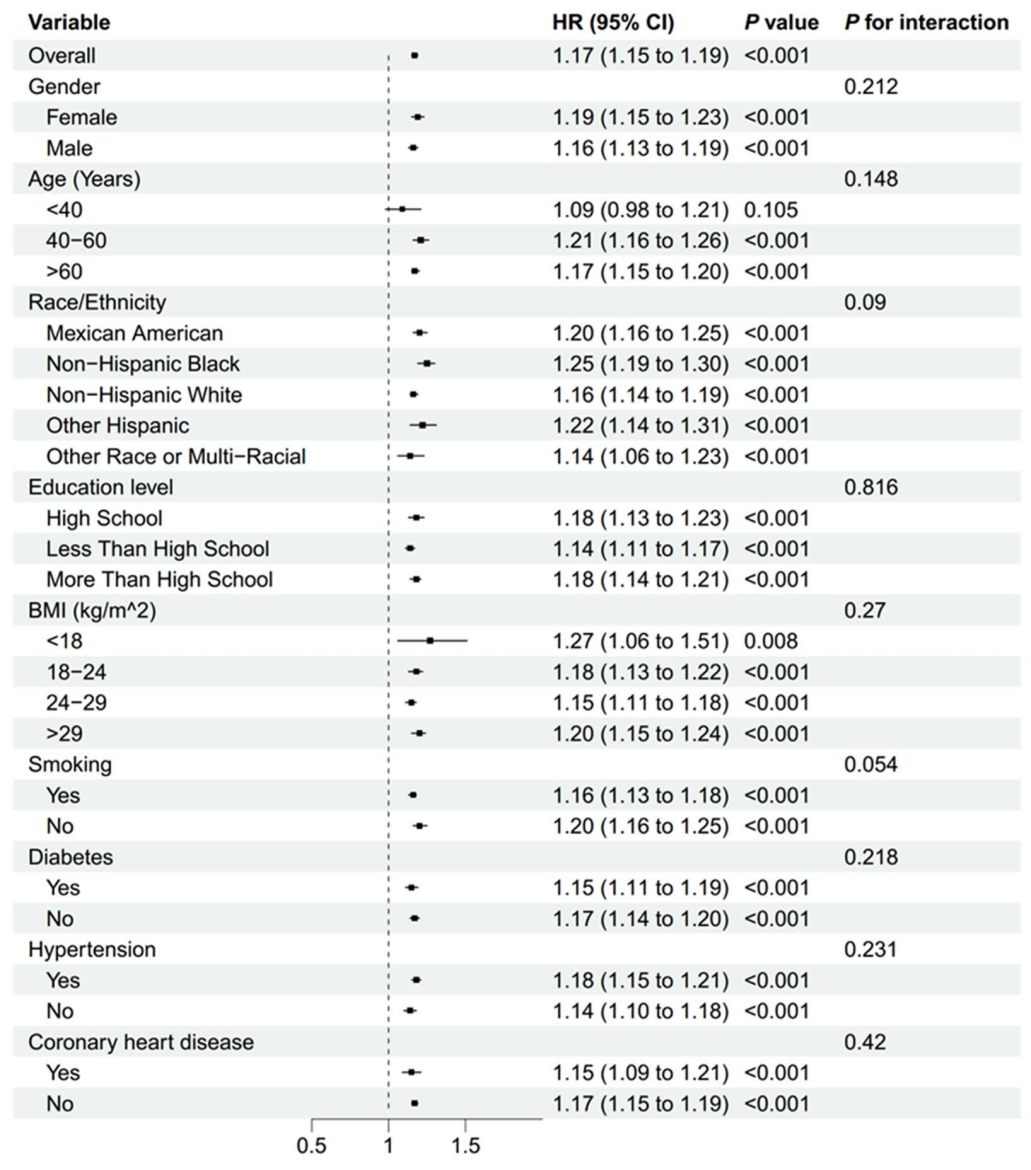
Subgroup analyses of the association between the NLR and all-cause mortality in all people. BMI, body mass index; NLR, neutrophil-to-lymphocyte ratio.

**Figure 7:**
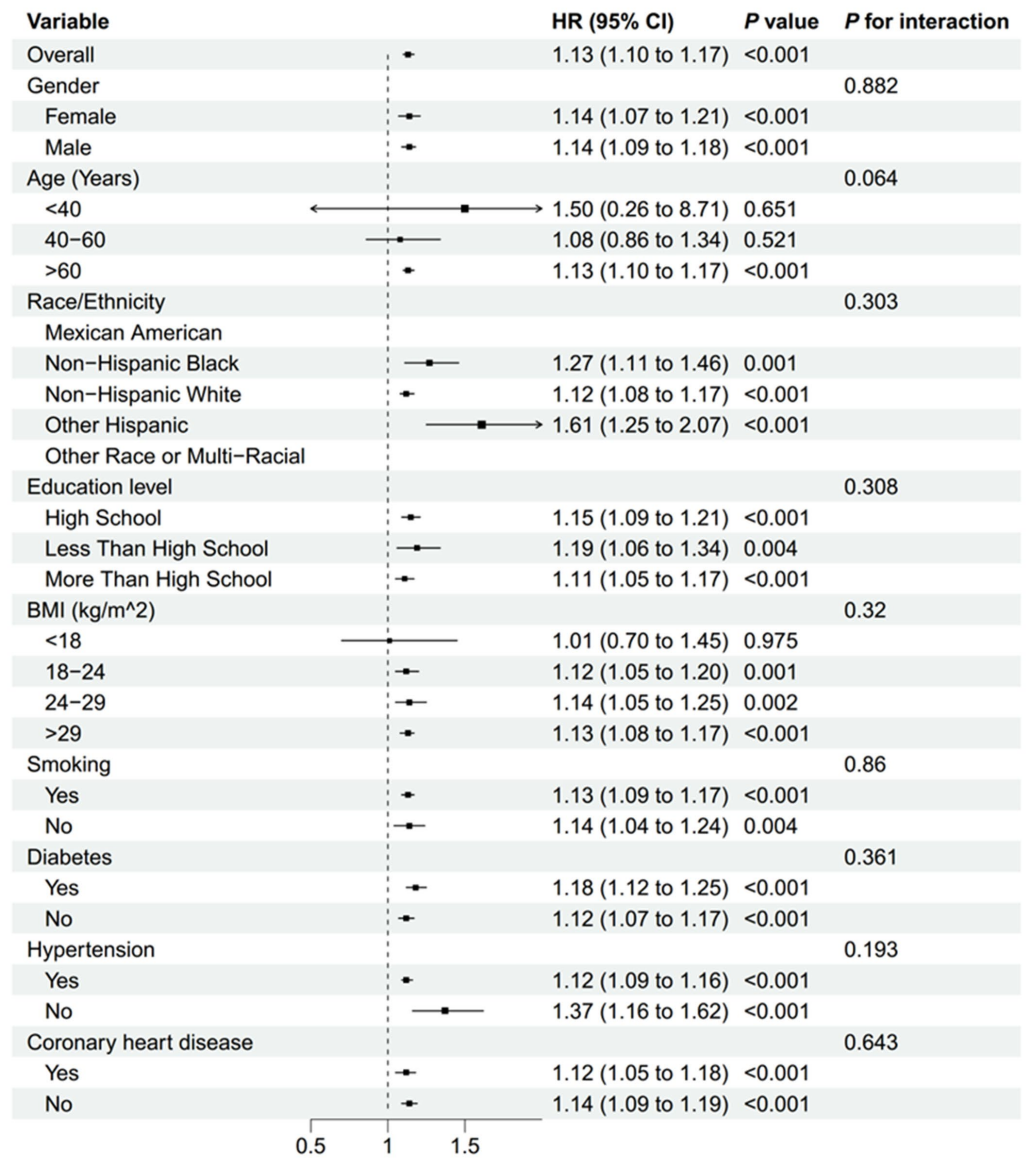
Subgroup analyses of the association between the NLR and all-cause mortality in patients with stroke. BMI, body mass index; NLR, neutrophil-to-lymphocyte ratio.

Subgroup analyses, presented in Figures 6, 7, demonstrated that the relationship between NLR and mortality was consistent across various subgroups in both the overall population and among stroke patients. No significant interactions were observed between NLR and the stratified variables, as indicated by all interaction *p*-values being greater than 0.05. Supplementary Table 1 provided detailed information on the time intervals between NLR examination, stroke and mortality.

## Discussion

In this study, we found a positive correlation between NLR and stroke prevalence. Additionally, NLR was positively associated with all-cause mortality both in the general population and among stroke patients. Subgroup analyses further demonstrated similar trends across different demographic and clinical groups.

Previous studies have reported associations between NLR and various diseases, including cardiovascular disease (18), chronic obstructive pulmonary disease (19, 20), acute intracerebral hemorrhage (21), and subarachnoid hemorrhage (22), using diverse epidemiological approaches and target populations. It is widely recognized that components of the immune system are closely linked to the onset and progression of ischemic brain injury, particularly ischemic stroke, which is largely attributed to a chronic inflammatory state. This inflammation, triggered by infectious or non-infectious factors, leads to systemic and vascular inflammation, resulting in oxidative stress, endothelial dysfunction, vascular wall injury, platelet activation and aggregation, and ultimately, intravascular thrombosis (14, 23). Peripheral immune markers have been shown to correlate with changes in multiple cortical and subcortical regions and white matter tracts, supporting the hypothesis that neuroinflammation is a significant contributor to the etiology of various brain diseases (24).

NLR is a potential novel biomarker of the baseline inflammatory response and has been shown to be an important predictor of morbidity and mortality in acute ischemic stroke (25). Furthermore, NLR has been studied as a predictive marker for stroke-associated pneumonia in stroke patients (6, 26). Our study identified a positive association between NLR and both stroke morbidity and all-cause mortality in stroke patients. Consistent with our findings, a study of 4,854 participants revealed that those in the higher NLR quartiles had an increased risk of incident stroke compared to those in the lowest quartile. For patients with minor ischemic stroke or transient ischemic attack (TIA), high neutrophil counts and ratios were linked to increased risks of recurrent stroke, composite events, and ischemic stroke (5).

A retrospective study also found that stroke patients with poorer outcomes at discharge and 3 months were more likely to have elevated NLR both at discharge and 3 months post-stroke (27). In a study evaluating the outcomes of patients with large vessel occlusion treated with mechanical thrombectomy, each unit increase in NLR was independently associated with increased odds of symptomatic intracranial hemorrhage and 3-month mortality. This suggests that NLR may help identify target groups for testing adjunctive anti-inflammatory therapies (15). Another study of 1,069 patients with acute ischemic stroke treated with intravenous thrombolysis found that NLR was associated with early neurological deterioration after thrombolysis (28). Collectively, these findings suggest an association between NLR and the risk of stroke and all-cause mortality in stroke patients. This study also identified the clinical value of NLR in assessing these risks.

The potential mechanisms linking NLR with stroke morbidity include interactions with endothelial cells and platelets, as well as excessive activation of neutrophil extracellular traps (NETs) (29). Neutrophils can release tissue factors that induce vasoconstriction and platelet aggregation, thereby promoting thrombosis (30–32). Some reports suggest that brain-infiltrating neutrophils play a detrimental role in ischemic tissue injury, causing brain damage through the release of reactive oxygen species and inflammatory mediators (33). Increased neutrophil concentration may lead to enhanced expression of matrix metalloproteinase-9, a protein associated with blood-brain barrier (BBB) disruption and brain injury (34, 35). Disruption of the BBB occurs in the early stages of stroke and facilitates the infiltration of peripheral leukocytes into the injured brain (36). Guided by inflammatory cytokines and chemokines released by ischemic tissue, these peripheral leukocytes may also affect ischemic regions (9). Lymphocytes, on the other hand, play a key role in inflammation, with certain lymphocytes exhibiting neuroprotective effects (37). Although further investigation is needed to fully understand the inflammatory mechanisms involved in stroke, NLR measurement is cost-effective, rapid, and widely available, making it a practical tool for low-cost monitoring systems. NLR could potentially enhance our ability to stratify risk in minor ischemic stroke or TIA, providing valuable guidance for prevention and treatment.

The primary strength of this study is the use of 11 cycles of NHANES data, which represents a multiethnic and gender-diverse adult population in the United States, making our findings more generalizable to the broader population. The large dataset enabled subgroup analyses, and extensive adjustments were made to account for potential confounders. The results of this study corroborated the importance of inflammation in stroke development and emphasized the value of inflammatory responses for treating and preventing stroke. The findings suggest NLR as a simple, cost-effective biomarker for predicting both stroke risk and all-cause mortality, particularly in clinical settings. This positions NLR as a potentially valuable tool for early stroke identification, risk stratification, and outcome prediction. However, several key limitations should be acknowledged. First, the use of self-reported methods to assess disease presence is susceptible to recall bias, which may impact data interpretation. Second, the NHANES database does not differentiate between the two main types of stroke (ischemic or hemorrhagic) or stroke subtypes, and the heterogeneous etiologies may affect the potential association between NLR and stroke. Third, although we adjusted for several potential confounders in the multivariable models, NLR may still be influenced by other factors. Finally, the cross-sectional design of the study precludes the establishment of causality, and further validation is required in large-scale prospective studies to confirm these findings.

## Conclusion

This study, by investigating the association of NLR with stroke morbidity and mortality, has identified the potential clinical value of NLR in assessing the risk and severity of stroke. The findings supported the critical role of inflammation status in stroke.

## Data Availability

The original contributions presented in the study are included in the article/Supplementary material, further inquiries can be directed to the corresponding authors.

## Footnotes

^1^ https://www.cdc.gov/nchs/nhanes/index.htm
