# Supplementary figures and images for: Association of neutrophil-to-lymphocyte ratio with stroke morbidity and mortality: evidence from the NHANES 1999–2020
Source: Front Med (Lausanne). 2025 Apr 2;12:1570630. doi: 10.3389/fmed.2025.1570630 (PMC12000060; doi:10.3389/fmed.2025.1570630)

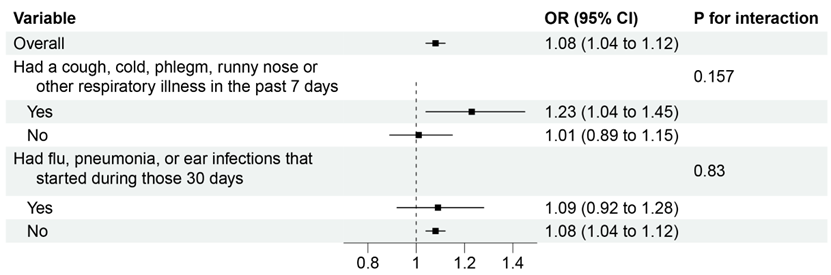

Supplement: Supplementary file 1 [file Image_1.tif]
