# Supplementary material for: Association of neutrophil-to-lymphocyte ratio with stroke morbidity and mortality: evidence from the NHANES 1999–2020
Source: Front Med (Lausanne). 2025 Apr 2;12:1570630. doi: 10.3389/fmed.2025.1570630 (PMC12000060; doi:10.3389/fmed.2025.1570630)
Supplement: Supplementary file 2 [file Table_1.docx]

**Supplementary Table 1. Time interval between NLR examination, stroke and mortality**

|  | years from first told had a stroke to NLR examination | years from NLR examination to follow-up endpoint |
| --- | --- | --- |
| stroke patients (died, N=736) | 8.48±10.40 | 6.33±4.22 |
| stroke patients (not died, N=692) | 8.65±9.09 | 9.54±4.47 |
| t | -0.322 | -13.977 |
| *P* | 0.747 | < 2.2e-16 |
